# The *Ustilago hordei*–Barley Interaction is a Versatile System for Characterization of Fungal Effectors

**DOI:** 10.3390/jof7020086

**Published:** 2021-01-27

**Authors:** Bilal Ökmen, Daniela Schwammbach, Guus Bakkeren, Ulla Neumann, Gunther Doehlemann

**Affiliations:** 1 BioCenter, Institute for Plant Sciences, University of Cologne, Zülpicher Straße 47a, 50674 Cologne, Germany; 2 Max Planck Institute for Terrestrial Microbiology, Karl von Frisch Straße, 35043 Marburg, Germany; Daniela.schwammbach@ukmuenster.de; 3 Summerland Research and Development Centre, Agriculture and Agri-Food Canada, Summerland, BC V0H 1Z0, Canada; guus.bakkeren@canada.ca; 4 Central Microscopy, Max Planck Institute for Plant Breeding Research, Carl-von-Linné-Weg 10, 50829 Cologne, Germany; neumann@mpipz.mpg.de

**Keywords:** *Ustilago hordei*, heterologous gene expression, effectors, haustoria, CRISPR-Cas9

## Abstract

Obligate biotrophic fungal pathogens, such as *Blumeria graminis* and *Puccinia graminis*, are amongst the most devastating plant pathogens, causing dramatic yield losses in many economically important crops worldwide. However, a lack of reliable tools for the efficient genetic transformation has hampered studies into the molecular basis of their virulence or pathogenicity. In this study, we present the *Ustilago hordei*–barley pathosystem as a model to characterize effectors from different plant pathogenic fungi. We generate *U. hordei* solopathogenic strains, which form infectious filaments without the presence of a compatible mating partner. Solopathogenic strains are suitable for heterologous expression system for fungal virulence factors. A highly efficient Crispr/Cas9 gene editing system is made available for *U. hordei*. In addition, *U. hordei* infection structures during barley colonization are analyzed using transmission electron microscopy, showing that *U. hordei* forms intracellular infection structures sharing high similarity to haustoria formed by obligate rust and powdery mildew fungi. Thus, *U. hordei* has high potential as a fungal expression platform for functional studies of heterologous effector proteins in barley.

## 1. Introduction

Plant pathogens have evolved different types of pathogenic lifestyles with their hosts, including obligate biotrophic, biotrophic, hemibiotrophic, and necrotrophic. For successful colonization, each pathogen deploys a distinct set of effectors that target specific host molecules, pathways, and structures. Recent genome and transcriptome analyses of a wide range of phytopathogens have provided new insights into the effector inventories of pathogens with different pathogenic lifestyles [1,2,3,4,5]. It has been found that compared to biotrophic pathogens, necrotrophs and hemibiotrophs have more plant cell wall degrading enzymes, secondary metabolites, and toxins in order to kill their host cells during the infection and feed on nutrients released from dead host cells [6,7,8]. On the other hand, effector catalogues of biotrophs appear to be more specialized, reflecting their ability to efficiently suppress host defenses, including regulated cell death, since their survival strictly depends on living host cells [7,8]. While many effectors from different facultative biotrophs, hemibiotrophs, and necrotrophs have been functionally characterized, there is still limited mechanistic insight into effectors of obligate biotrophic filamentous pathogens. A main reason for this gap is the absence of efficient genetic transformation and gene deletion techniques available to perform reverse genetics in obligate biotrophs.

Currently, the functional characterization of effectors of obligate biotrophic pathogens is performed using different strategies. Effectors from these pathogens can be heterologously expressed in planta and their positive contribution to virulence can be determined via subsequent inoculation of these plants with several pathogens [9,10]. However, heterologous expression of some effectors in planta can result in strong pleiotropic defects that compromise symptom evaluations. In another strategy, the type III secretion system (T3SS) of *Pseudomonas syringae* (for Arabidopsis) and *Pseudomonas fluorescens* or *Pseudomonas atropurpurea* (for wheat and barley) is used for functional characterization of several intracellular effectors from obligate biotrophs, such as rusts and powdery mildews [11,12,13,14,15]. Any growth promotion observed for *Pseudomonas* sp. transformants, which deliver the desired effectors into the host cell during infection, is interpreted as a positive contribution to virulence [11,12,13,14,15]. However, the T3SS of *Pseudomonas* sp. also has some drawbacks. For example, fungal effectors that require posttranslational modifications for their activity will not be correctly produced by the *Pseudomonas* sp. system, since prokaryotes lack the molecular machinery necessary for these modifications. In addition, the T3SS system delivers effectors into host plant cells, and hence fungal effectors that play roles in the apoplast or are required for haustorium formation and function during host colonization might not be identified. Furthermore, the function of some effectors from biotrophic pathogens is to avoid or suppress PAMP-triggered immunity (PTI) to promote disease establishment. PAMPs from bacterial and fungal pathogens are different because of their phylogenetic distance, so unless the signaling pathways that lead to PTI are completely conserved, PTI responses induced by *Pseudomonas* sp. may not be evaded or suppressed by fungal effectors. In another method, to validate virulence function of obligate biotroph effector genes during host colonization, a host-induced gene silencing (HIGS) assay was developed [16,17,18]. However, the requirements for stable transgenic host lines of HIGS constructs make this method very laborious.

The facultative biotrophic fungal pathogen *Ustilago hordei* is a causal agent of covered smut disease on barley and oat plants. *U. hordei* belongs to the group of Ustilaginales, members of which infect many economically important crops, including maize, wheat, barley, oat, and sugar cane. Similar to other smut fungi, pathogenic development of *U. hordei* is coupled to sexual development [19]. For successful infection, two haploid sporidia of opposite mating-types fuse to form an infectious dikaryotic filament, which subsequently differentiates to form an appressorium, a swollen hyphal cell that leads to direct penetration of host epidermal cells. During plant colonization, *U. hordei* proliferates both extra- and intracellularly and forms haustorium-like feeding structures in the host cells [20]. *U. hordei* reaches and establishes itself in the host meristem and then grows with the plant until the floral meristem develops spikelets, which likely gives a cue to the fungus to multiply and sporulate. Massive proliferation and sporulation of the fugus in the barley inflorescence is displayed by mass production of dark brown smut teliospores [21].

*Blumeria graminis* f. sp. *hordei* (*Bgh*) and *Puccinia graminis* f. sp. *tritici* (*Pgt*) are obligate biotrophic pathogens that are the causal agents of powdery mildew and stem rust on barley, respectively [22,23]. Unlike *U. hordei*, which can be cultured in vitro, both *Bgh* and *Pgt* have obligate biotrophic lifestyles and cannot be cultured outside the host. Therefore, the generation of stable fungal transformants is the main bottleneck to studying these pathosystems at the molecular level. Despite their phylogenetic distance, *Bgh* (Ascomycota), *Pgt*, and *U. hordei* (Basidiomycota) share significant similarities—they are barley or wheat pathogens and they establish strictly biotrophic interactions with their host, in which they form specialized intracellular feeding structures, the haustoria [20,22,23]. These similarities prompted us to establish cell biological, molecular, and genetic methods to use the *U. hordei*-barley interaction as a model system for functional characterization of effector candidates from different filamentous phytopathogens.

## 2. Material and Methods

### 2.1. Plant and Fungal Materials

To isolate total genomic DNA from axenic culture, *Ustilago hordei* (4857-4) strains were incubated in YEPS_light_ (0.4% yeast extract, 0.4% peptone, and 2% saccharose) liquid medium at 22 °C with 200 rpm shaking till OD_600nm_:1.0. To isolate total gDNA from *U. hordei*-infected barley plants at 6 days post inoculation (dpi), the third leaves of the *U. hordei*-infected barley plants were collected by cutting 1 cm below the injection needle sites. Leaf samples were then frozen in liquid nitrogen and ground using a mortar and pestle under constant liquid nitrogen. The gDNA was isolated by using a MasterPure™ Complete DNA and RNA Purification Kit (Epicentre^®^, Illumina^®^, Madison, WI, USA) according to the manufacturer’s instructions.

Susceptible Golden Promise barley cultivar was grown in a greenhouse at 70% relative humidity at 22 °C during the day and at night, with a light/dark regime of 15:9 h (hours) and with 100 Watt m^−2^ supplemental light when the sunlight influx intensity was less than 150 Watt m^−2^.

### 2.2. Nucleic Acids Methods

Fungal biomass quantification was performed by using quantitative PCR (qPCR) analysis as in Ökmen et al. [20]. Genomic DNA from infected barley leaves at 6 days post inoculation (dpi) was isolated by using the MasterPure™ Complete DNA and RNA purification Kit (Epicentre^®^, Illumina^®^) according to manufacturer’s instructions. The *U. hordei UhPpi* gene (*UHOR_05685*) was used as a reference gene. A standard curve was constructed by using serial dilutions of *U. hordei* genomic DNA (100, 10, 1, 0.1, 0.01, 0.001 ng µL^−1^) using *UhPpi* as a reference gene. Base 10 logarithms of DNA concentrations were plotted against the crossing point of Ct values. The qPCR reaction was performed in a Bio-Rad iCycler system by using the following program: 2 min at 95 °C followed by 45 cycles of 30 s at 95 °C, 30 s at 61 °C, and 30 s 72 °C. The primers that were used for qPCR are listed in Table S1.

All PCR reactions were performed by using Phusion© DNA polymerase (Thermo Scientific; Bonn, Germany) following the manufacturer’s instructions, with 100 ng genomic DNA or cDNA as the template. All primers that were used in PCR reactions for cloning of different genes are listed in Table S1. The amplified DNA fragments were then used for cloning processes. All PCR reaction took place in a PTC-200 (Peletier Thermal Cycler, Bio-Rad MJ Research, Hercules, CA, USA) PCR machine. Nucleic acids derived from PCRs or restriction digests reactions were purified from 1% TAE (Tris-Acetate-EDTA) agarose gels with the Wizard SV Gel and Purification System Kit (Promega) according to the manufacturer’s instructions. Plasmid isolation from bacterial cells was performed using the QIAprep Mini Plasmid Prep Kit according to the manufacturer’s instructions.

### 2.3. Construction of Expression Vectors

For heterologous gene expression constructs (*p*123-*pUHOR02700*::*SP*-*Gus*-*mCherry*, *p123*-*pUHOR*02700::*Gus*-*mCherry*, *p*123-*pUHOR*02700::*SP*-*FvRibo*1, and *p*123-*pActin*::*SP*-*CfAvr*4), standard molecular biology methods were used according to the molecular cloning laboratory manual of Sambrook et al. [24]. Amplified PCR fragments for each gene (*Gus-mCherry*, *FvRibo*1, and *CfAvr*4) were cut with appropriate restriction enzymes, then subsequently they were ligated into a vector that was digested with the same restriction enzymes by using T4-DNA ligase (New England Biolabs; Frankfurt a.M., Germany) according to manufacturer’s instructions. The sequence confirmation of each construct was performed via sequencing at Eurofins Genomics (Cologne, Germany). All vector constructs, primer pairs, and restriction sites are shown in Table S1. *Escherichia coli* transformation was performed via heat shock assay according to standard molecular biology methods [24].

### 2.4. CRISPR/Cas9 Gene Editing System

To establish the CRISPR/Cas9-HF (high fidelity) gene editing system in *U. hordei*, a plasmid containing codon-optimized *Cas9-HF* genes under the control of *Hsp70* promoter and carboxin resistance was used according to the method of Zuo et al. [25]. To express sgRNA for targeted genes, the *Ustilago maydis pU6* promotor was replaced with the *U. hordei pU6* promotor. The sgRNA for the knockout of the *U. hordei* gene was designed by E-CRISPR (http://www.e-crisp.org/ECRISP/aboutpage.html) (Table S1) [26]. Plasmid construction for CRISPR/Cas9 was performed as described by Zou et al. [25]. The CRISPR/Cas9-HF vector was linearized with restriction enzyme Acc65I and subsequently assembled with an oligo spacer and scaffold RNA fragment with 3’ downstream 20 bp overlap to the plasmid by using the Gibson assembly method [27]. To test the efficiency of our CRISPR/CAS9 gene editing assay in *U. hordei* DS200, a homologous gene of *U. maydis Fly1* (encoding a secreted metalloprotease) was used as a target for CRISPR/CAS9. Deletion of *Fly1* in *U. maydis* resulted in an impaired cell separation phenotype. After the *U. hordei Fly1* gene was edited via CRISPR/Cas9 mutagenesis, resulting in a truncated protein (Table S1), impaired cell separation phenotype of the DS200Δfly1 mutant was observed under a microscope to determine the efficiency of the CRISPR/CAS9 gene editing assay.

### 2.5. Fungal Transformation and Virulence Assays

The *U. hordei* transformation assay was conducted by using protoplasts according to Kämper [28]. Virulence assays for DS200-FvRibo1 and DS200 *U. hordei* strains were performed according to Ökmen et al. [20]. Briefly, all *U. hordei* strains were grown in YEPS_light_ liquid medium at 22 °C with 200 rpm shaking until reaching an optical density (OD_600_) of 0.6–0.8. Subsequently, *U. hordei* cells were centrifuged at 3500 rpm for 10 min at RT (room temperature) and resuspended in sterile distilled water supplemented with 0.1% Tween 20 to an OD_600_ of 3.0. Then, each *U. hordei* cell suspension was injected into stems of 12-day-old barley seedlings (Golden Promise) with a syringe with a needle. All infection assays were performed in three biological replicates, with at least 15 plants used for each replicate. Fungal biomass quantification of *U. hordei* was performed at 6 days post inoculation (dpi) by using genomic DNA (200 ng µL^−1^) as the template with qPCR. To confirm secretion of GUS-mCherry protein in the apoplast of barley leaf, apoplastic fluid (AF) from *U. hordei* DS200 ± GUS-mCherry strain infected barley leaves were isolated according to van der Linde et al. [29]. After AF isolation, Western blot analysis was performed to detect GUS-mCherry signals in isolated AFs. Western blot analysis was performed as described in Mueller et al. [30]. To confirm that *U. hordei* can express and secrete functional effector proteins from different fungi, *Avr4* of *Cladosporium fulvum* was heterologously expressed in the *U. hordei* strain DS200 with the *UHOR_02700* signal peptide (SP) and under control of the *pActin* promoter to produce the protein in axenic culture (in vitro expression); and heterologously expressed together with *Ribo1* (encoding a secreted ribotoxin) of *Fusarium verticillioides* in the *U. hordei* strain DS200 with the *UHOR_02700* signal peptide (SP) and under control of the *pUHOR_02700* promoter for in planta expression. To confirm expression and secretion of CfAvr4 effector protein in *U. hordei* in vitro, culture filtrates isolated from DS200-CfAvr4, DS200-FvRibo1, and DS200 strains (OD_600_:1.0) were collected with centrifugation (13,000 rpm for 5 min). After filter sterilization, each culture filtrate was infiltrated in tobacco leaves expressing *Cf4* resistance gene (a gene encoding tomato Cf4 receptor protein, which can recognize CfAvr4 protein and induce hypersensitive response) to induce a hypersensitive response (HR).

### 2.6. Light Microscopy

The wheat germ agglutinin (WGA)-AF488 (Molecular Probes, Karlsruhe, Germany) and propidium iodide (PI) (Sigma-Aldrich) staining was performed according to Ökmen et al. [20]; WGA-AF488 stains fungal cell walls and propidium iodide stains plant cell walls. *U. hordei*-infected barley leaves were first bleached in pure ethanol and then boiled for 1–2 h in 10% KOH at 85 °C. Subsequently, the pH of boiled leaf samples was neutralized by using 1× PBS buffer (pH: 7.4) with several washing steps. Then, the WGA-AF488/PI staining solution (1 µg mL^−1^ propidium iodide, 10 µg mL^−1^ WGA-AF488; 0.02% Tween 20 in PBS pH 7.4) was vacuum infiltrated in samples for 5 min at 250 mbar using a desiccator (vacuum infiltration step was performed three times). The WGA-AF488/PI-stained leaf samples were stored in 1× PBS buffer (pH: 7.4) at 4 °C in the dark until microscopy. WGA-AF488: excitation at 488 nm and detection at 500–540 nm. PI: excitation at 561 nm and detection at 580–630 nm.

To visualize secretion of GUS-mCherry protein in *U. hordei* during barley colonization, *U. hordei* DS200 ± GUS-mCherry strains were inoculated on barley plants. Subsequently, infected barley leaves were checked for localization of GUS-mCherry at 4 dpi by using a Leica SP8 confocal microscopy. For mCherry fluorescence of hyphae in barley tissue, excitation at 561 nm and detection at 580–630 nm were performed.

### 2.7. Transmission Electron Microscopy

Chemically fixed samples were prepared according to Wawra et al. [31] with minor changes. For TEM observation, 2 mm leaf discs from infected and non-infected *Hordeum vulgare* leaves were excised from 1 cm below infection sites by using a biopsy punch and chemically fixed in 2.5% glutaraldehyde and 2% paraformaldehyde in 0.05 M sodium cacodylate buffer, pH 6.9, supplemented with 0.025% CaCl_2_ (*w*/*v*) for 2 h at room temperature. Subsequently, samples were rinsed six times for 10 min in 0.05 M sodium cacodylate buffer (pH 6.9, rinses 3 and 4 supplemented with 0.05 M glycine), then postfixed for 1 h at room temperature with 0.5% OsO_4_ in 0.05 M sodium cacodylate buffer, pH 6.9, supplemented with 0.15% potassium ferricyanide. After thorough rinsing in 0.05 M sodium cacodylate buffer (pH 6.9) and water, samples were dehydrated in an ethanol series from 10% to 100%, gradually transferred to acetone, then embedded into Araldite 502/Embed 812 resin (EMS, catalog number 13940) using the ultra-rapid infiltration by centrifugation method used by McDonald [32].

For TEM observation, leaf samples were also processed by means of high-pressure freezing and freeze substitution as an alternative to conventional chemical fixation following the procedure described by Micali et al. [33] for ultrastructural observations [33]. Once the samples reached room temperature, they were rinsed in acetone, carefully removed from the aluminum specimen carriers, then gradually infiltrated in LR White resin (Plano GmbH) for 6 days. Resin polymerization was done in flat embedding molds at 100 °C for 24 h.

Ultrathin (70–90 nm) sections were collected on nickel slot grids as described by Moran and Rowley [34], stained with 0.1% potassium permanganate in 0.1 N H2SO4 [35], followed by 2% (*w*/*v*) aqueous uranyl acetate and lead citrate for 15 min [36], then examined with an Hitachi H-7650 TEM (Hitachi High-Technologies Europe GmbH, Krefeld, Germany) operating at 100 kV fitted with an AMT XR41-M digital camera (Advanced Microscopy Techniques, Danvers, MD, USA). Immunogold labeling of ß-1,3-glucan was done according to the procedures described previously [33].

## 3. Results

### 3.1. Construction of Solopathogenic Strain of Ustilago hordei

**Figure 1:**
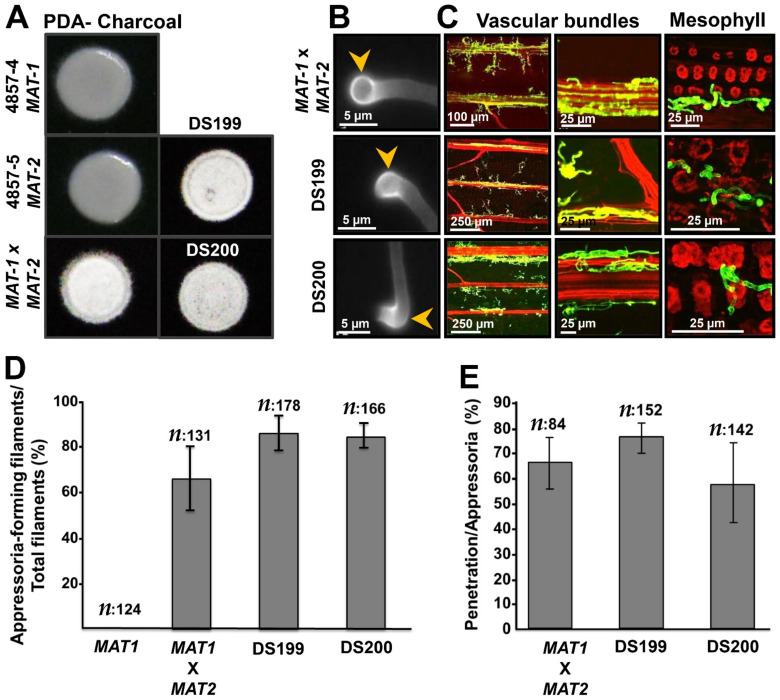
Generation of a solopathogenic *Ustilago hordei* strain. (**A**) Filamentation test on charcoal plate. *U. hordei* wild-type strains 4857-4 *MAT-1*, 4857-5 *MAT-2*, mating of 4857-4 *MAT-1* × 4857-5 *MAT-2*, solopathogenic DS199, and DS200 strains. Pictures were taken after 3 days incubation at RT. (**B**) Appressoria formation ability of *U. hordei* strains on parafilm. Mating of *U. hordei* wild-type 4857-4 *MAT-1* and 4857-5 *MAT-2*, solopathogenic DS199, solopathogenic DS200. Yellow arrowheads indicate appressoria. Pictures were taken after 24 h (hours) incubation (**C**) Disease development of different *U. hordei* strains *on* barley. Mating of *U. hordei* wild-type 4857-4 *MAT-1* and 4857-5 *MAT-2* at 3 dpi (days post inoculation), solopathogenic DS199 at 3 dpi, solopathogenic DS200 at 3 dpi. Following wheat germ agglutinin (WGA)-AF488/propidium iodide (PI) staining, fungal cell walls are shown in green and plant cell walls in red. (**D**) Quantification of appressoria formation for *U. hordei* wild-type 4857-4 *MAT1*, solopathogenic DS199, and DS200 on plant. (**E**) Quantification of penetration efficiency for *U. hordei* wild-type 4857-4 *MAT1*, solopathogenic DS199, and DS200 on plant.

The requirement of mating for the induction of pathogenic development implies that genetic mutations always need to be made in both compatible *U. hordei* strains, which presents an obvious drawback of the system, particularly for larger scale analyses. To optimize the work flow, we generated a solopathogenic *U. hordei* strain, which does not require a mating partner to form an infectious filament (Figure 1A–D). For pathogenic development, *U. hordei* requires both a compatible pheromone (Mfa)/pheromone receptor (Pra) pair and an active heterodimer made from *bE* and *bW* gene products [37,38,39]. To construct solopathogenic *U. hordei* strains, the *bE1* alleles from mating-type locus 1 (*MAT-1*) were replaced with the *bE2* allele from mating-type locus 2 (*MAT-2*), or both *Mfa1* and *bE1* alleles from mating-type locus 1 (*MAT-1*) were replaced with *Mfa2* and *bE2* alleles from mating-type locus 2 (*MAT-2*). While the constructed DS199 solopathogenic strain had a compatible *b*-locus with *bE2* and *bW1* genes from different mating-types, the DS200 solopathogenic strain contained both compatible MFA2/PRA1 and bE2/bW1 pairs to facilitate the formation of infectious filaments in the absence of a mating partner. For generation of DS199 and DS200 strains, homologous recombination constructs with an FRT [flippase (FLP) recombinase target]-flanked hygromycin resistance cassette (for *Mfa2* construct) or phleomycin resistance cassette (for *bE2* construct) were used, which were removed from the genome after induction of the FLP recombinase [40,41].

Both the DS199 and DS200 solopathogenic strains were then used for further analysis. To show the filamentation ability of solopathogenic strains, single *U. hordei* 4857-4 *MAT-1* and 4857-5 *MAT-2* strains, mixed 4857-4 *MAT-1* × 4857-5 *MAT-2*, and solopathogenic strains were grown on PDA (potato dextrose agar) plates containing charcoal. While single 4857-4 *MAT-1* and 4857-5 *MAT-2*A strains were not filamentous, mated 4857-4 *MAT-1* × 4857-5 *MAT-2* and solopathogenic strains were fully filamentous on PDA charcoal plates (Figure 1A). To test whether the solopathogenic strains form infection structures (hyphal tip swellings; appressoria) that are required for host penetration, mated wild-type *U. hordei* 4857-4 *MAT-1* and 4857-5 *MAT-2*, as well as solopathogenic strains, were sprayed on parafilm, which previously has been shown to artificially induce appressorium formation in *Ustilago maydis* [42]. This showed that both solopathogenic strains form appressoria are comparable to the wild-type strain (Figure 1B). Quantification of appressoria formation on barley leaves revealed that there are no significant differences in appressoria formation (Figure 1D) and penetration efficiency (Figure 1E) in wild-type and solopathogenic strains. Wheat germ agglutinin-AF488/propidium iodide (WGA-AF488/PI) staining of wild-type and solopathogenic strains infecting barley leaves revealed that there is no visible difference in colonization at 3 days post inoculation (dpi), i.e., all three strains were found to colonize the host mesophyll tissue (Figure 1C). While the leaf colonization assay did not show obvious differences between solopathogenic and wild-type strains, in barley seed infection assays, the solopathogenic strain (DS200) was rarely found to have colonized the barley inflorescence or produce teliospores (after 3–4 months post inoculation) (Figure S1).

### 3.2. Ultrastructure of the Ustilago hordei–Barley Interphase during Biotrophic Interaction

**Figure 2:**
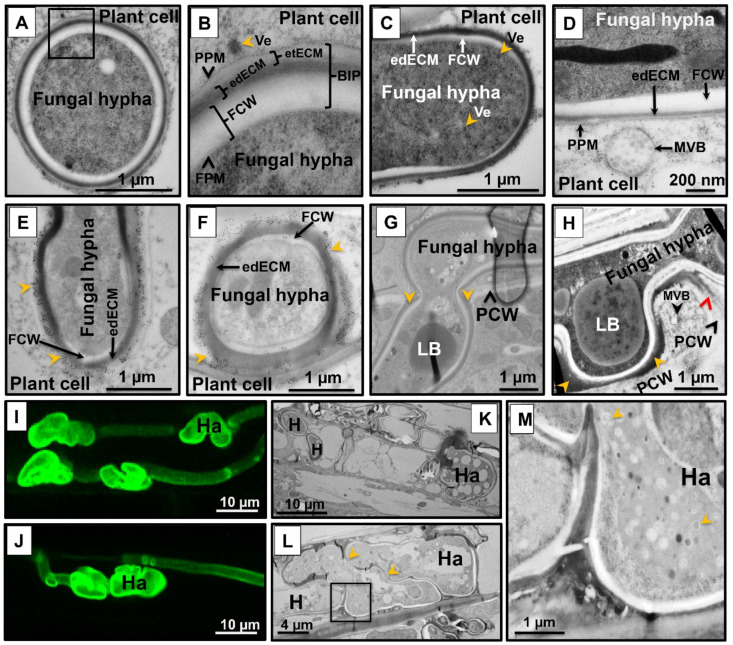
(**A**–**H**) Transmission electron microscopy micrographs of wild-type *Ustilago hordei*-infected barley leaves. (**A**,**B**) Biotrophic interphase in the *U. hordei*–barley interaction. During host colonization, *U. hordei* invaginates the host cell membrane without breaching it. The host–pathogen interaction mainly takes place within this biotrophic interphase (BIP), which consists of the fungal cell wall (FCW), electron-dense extracellular matrix (edECM), and electron-translucent extracellular matrix (etECM). (**C**,**D**) Formation of vesicles at the hyphal tip of *U. hordei*. Fungal vesicles (Ve) with cores of different electron densities and plant multivesicular bodies (MVB) were detected at hyphal tips and in the plant cytoplasm close to fungal penetration sites, respectively. (**E**,**F**) Immunogold labeling of callose with a monoclonal antibody recognizing (1-3)-β-glucan epitopes. Callose accumulation was detected at the electron-translucent ECM (etECM) site (yellow arrowheads). (**G**,**H**) Cell-to-cell penetration of *U. hordei*. *U. hordei* primarily grows intracellularly at 8 dpi in barley leaves. When the fungal hyphae penetrate a new plant cell, the hypha gets thickened at the site of cell-to-cell passage, resembling appressorial structures (**G**). The edECM gets thicker at the site of hypha contact with the plant cell wall (yellow arrowheads) (**H**), while electron-dense material can also diffuse into adjacent parts of the plant cell wall (red arrowhead) (**H**). (**I**–**M**) Haustoria formation during host colonization. *U. hordei* grows intracellularly and forms haustorial structures in barley cells. (**I**,**J**) Wheat germ agglutinin (WGA)-AF488/propidium iodide (PI) staining was performed to visualize *U. hordei* at 8 days post inoculation (dpi) under confocal/fluorescent microscopy. (**K**–**M**) Transmission electron micrographs showing different planes of the section through haustoria. Haustorial structures were distinguished from hyphae by their bigger size and interconnected lobular shapes. Yellow arrowheads (**L**) point out the connections between haustorial lobes. *U. hordei* haustoria possess large vacuoles with a granular lumen containing vesicles of different sizes (**M**) (yellow arrowheads; magnification of inset in (**L**)). BIP: biotrophic interphase; FCW: fungal cell wall; FPM: fungal plasma membrane; H: hypha; Ha: haustorium; edECM: electron-dense extracellular matrix; etECM: electron-translucent extracellular matrix; LB: lipid bodies; MVB: multi-vesicular body; PCW: plant cell wall; PPM: plant plasma membrane; Ve: vesicles.

Similar to powdery mildews and rusts, smut fungi including *U. hordei* are considered to be intracellular pathogens. However, in all of these interactions, the host plasma membrane is not breached, and thus the host–pathogen interaction takes place through the biotrophic interphase, which consists of the fungal cell wall (FCW), extracellular matrix (ECM), and plant cell wall (in some regions) (Figure 2A,B). Both chemically fixed and high-pressure-frozen samples were used to perform transmission electron microscopy (TEM) to display ultrastructural features of the wild-type *U. hordei*–barley biotrophic interphase during infection. Samples were taken at 8 dpi, when *U. hordei* growth is primarily intracellular and hyphae can be found in epidermal cells, mesophyll cells, and vascular bundles. Transmission electron micrographs show fungal hyphae growing inside plant cells and frequently branching (Figure S2A–D). The fungal hyphae contain free ribosomes; strands of endoplasmic reticulum; mitochondria; nuclei; as well as lipid bodies, vesicles, and vacuoles (Figure S3A–F). Transmission electron micrographs also show the presence of closely paired nuclei in fungal hyphae, which are intimately associated with mitochondria (Figure S3E). Vesicles and multivesicular bodies were also frequently observed at hyphal tips and in the plant cytoplasm adjacent to fungal penetration sites, respectively (Figure 2C,D and Figure S3B,C,F).

It caught our attention that the *U. hordei* hyphal cell wall appears to be surrounded by a two-layered extracellular matrix of different electron densities—an inner electron-dense extracellular matrix (edECM) layer and an outer electron-translucent extracellular matrix (etECM) layer, both of unknown composition (Figure 2A,B and Figure S3A–C). Immunogold labeling with a monoclonal antibody specific for (1-3)-β-glucans showed that callose is present in the etECM (Figure 2E,F). The edECM was particularly prominent (between 50–500 nm thickness) when the hyphae were in contact with the plant cell wall (Figure 2H, yellow arrowheads, Figure S2A,D). In some areas, the middle lamella of the plant cell wall close to the interface was more electron-dense than in areas where it was not in contact with the fungal hypha (Figure 2H, red arrowhead). Another interesting observation of our TEM analysis was that at the site of cell-to-cell penetration, the *U. hordei* hypha is swollen, resembling appressorial structures (Figure 2G and Figure S2C). During barley colonization, *U. hordei* also forms structures similar to the haustoria known for obligate biotrophs, where they are described to function as feeding structures (Figure 2I–M). Haustorial structures of *U. hordei* were distinguished from the normal hyphae by their bigger size and interconnected lobular shapes (Figure 2I–L). High magnification transmission electron micrographs of *U. hordei* haustoria showed that these structures possess large vacuoles with a granular lumen containing vesicles of different sizes (Figure 2M).

### 3.3. Heterologous Gene Expression in Ustilago hordei

**Figure 3:**
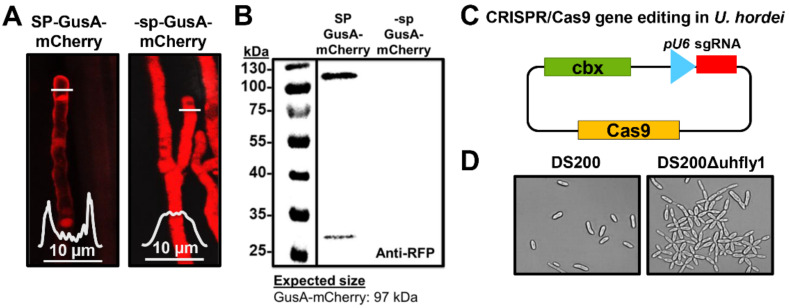
(**A**,**B**) Heterologous expression of *GusA-mCherry* in *Ustilago hordei*. (**A**) GusA-mCherry was heterologously expressed in solopathogenic strain DS200 under control of the *UHOR_02700* promotor with or without signal peptide (SP) for extracellular secretion. The ± SP-GusA-mCherry DS200 strains were inoculated on barley seedlings, then at 4 days post inoculation (dpi) confocal microscopy was performed to monitor expression and localization of recombinant proteins. While +SP-GusA-mCherry is secreted around the tip of the invasive hyphae, -sp-GusA-mCherry localizes in the fungal cytoplasm. The white graphs indicate the mCherry signal intensity along the diameter of the hyphae (illustrated by white lines in the image). (**B**) Western blot analysis was performed with apoplastic fluid isolated from barley leaves infected with ± SP-GusA-mCherry DS200 strains. While a band corresponding to secreted +SP-GusA-mCherry (at ~100 kDa) and free mCherry (at 27 kDa) in isolated apoplastic fluid was detected, no band corresponding to cytoplasmic -sp-GusA-mCherry could be detected. Anti-RFP antibody was used for Western blot analysis. (**C**,**D**) Establishment of CRISPR/Cas9 gene editing system for *Ustilago hordei*. (**C**) Codon-optimized *Cas9* was cloned into the *p123* plasmid under the control of the *Hsp70* promoter. The *U. hordei pU6* promotor was used to express sgRNA for the targeted gene. Carboxin resistance was used as selection marker. (**D**) *U. hordei Fly1* gene, a fungalysin metalloprotease involved in fungal cell separation, was edited via the CRISPR/Cas9 system for knock-out. While DS200 sporidia showed normal growth, DS200Δuhfly1 cells were impaired in cell separation.

Regarding the use the *U. hordei*–barley pathosystem for functional characterization of secreted virulence factors, heterologous gene expression was established in this smut fungus. As a proof of concept, *mCherry* fused to the *Escherichia coli GusA* gene under the control of the *U. hordei UHOR_02700* promoter (highly induced upon barley penetration) was heterologously expressed in the *ip* (*cbx*) locus of the solopathogenic DS200 strain, either with (+) or without (−) the signal peptide (from *UHOR_02700*) for extracellular secretion (Figure 3A). Confocal microscopy imaging was performed with DS200 strains expressing ± SP-GusA-mCherry on barley leaves at 3 dpi to monitor expression and localization of recombinant proteins. While SP-GusA-mCherry was localized around the hyphal tip region (showing secretion from the biotrophic hypha), -sp-GusA-mCherry was localized inside the fungal cytoplasm (Figure 3A). Furthermore, Western blot analysis was performed with apoplastic fluid isolated from barley leaves infected with ± SP-GusA-mCherry DS200 strains to confirm secretion of the recombinant proteins. Western blot results also showed that while the secreted full-length SP-GusA-mCherry (~100 kDa) and cleaved free mCherry (~27 kDa) were detected in isolated apoplastic fluid, the cytoplasmic -sp-GusA-mCherry was not detectable in isolated apoplastic fluid (Figure 3B).

### 3.4. CRISPR/Cas9 Gene Editing in Ustilago hordei

To establish the CRISPR/Cas9-HF (high fidelity) gene editing system in *U. hordei*, a codon-optimized *Cas9*-*HF* gene under the control of the *Hsp70* promoter was expressed in the solopathogenic strain DS200. The *U. hordei pU6* promotor was used to express the sgRNA of a targeted gene (Figure 3C). As a proof of concept, the *U. hordei Fly1* gene, a fungalysin metalloprotease involved in fungal cell separation in *U. maydis* [43], was edited via the CRISPR/Cas9 system to result in a truncated protein (after aa 25, a stop codon was introduced via generation of error in the gene). Microscopic observations showed that while the DS200 strain formed normal yeast cells, DS200Δfly1 strains were impaired in cell separation in liquid medium, indicating functional conservation of Fly1 in both *U. hordei* and *U. maydis* (Figure 3D). In total, 83.3% (±8.3%) of selected independent transformed colonies showed an impaired cell separation phenotype, indicating the high efficiency of this method in *U. hordei*.

### 3.5. Activity of Heterologous Virulence Factors Expressed in Ustilago hordei

**Figure 4:**
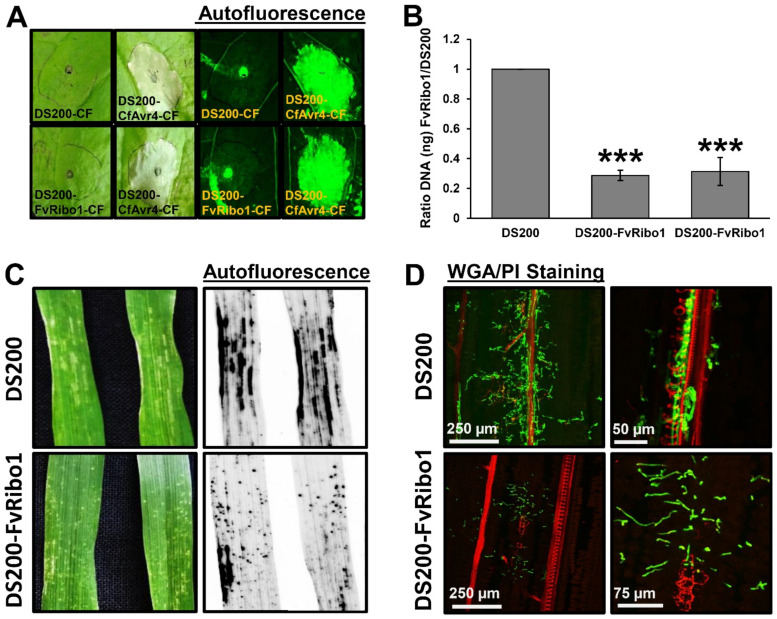
Heterologous expression of fungal effectors in *Ustilago hordei*. (**A**) Heterologous expression and secretion of CfAvr4 in *U. hordei* DS200 strain in vitro. *U. hordei* strain DS200 expressing *CfAvr4* of *Cladosporium fulvum* with *UHOR_02700* signal peptide and under the control of *pActin* promoter (for constitutive expression), as well as FvRibo1 of *Fusarium verticillioides* with *UHOR_02700* signal peptide and under the control of *pUHOR_02700* promoter (for expression in planta only) were grown in YEPS_light_ liquid medium till OD:1.0. The *U. hordei* cell suspensions were centrifuged and the culture filtrates (CF) of each sample were infiltrated into tobacco leaves expressing Cf4-resistant protein, which can recognize CfAvr4 and induce cell death by means of hypersensitive response (HR). The culture filtrates from *U. hordei* DS200 and DS200-FvRibo1 strains were used as negative controls. Pictures were taken at 5 days post infiltration (dpi). Autofluorescence of infected leaves was imaged to more easily see sites of cell death by using Gel-Doc (Bio-Rad). (**B**) Biomass quantification of DS200-FvRibo1 in barley leaves. The virulence of the *U. hordei* DS200 and two independent DS200-FvRibo1 strains was assessed by fungal biomass quantification from DNA isolated from infected barley leaves at 6 days post inoculation (dpi). The *Ppi1* gene of *U. hordei* was used as a standard for qPCR. The fungal biomass was deduced from a standard curve. A student t-test was performed to determine significant differences, which are indicated as asterisks (***, *p* < 0.001). Error bars represent the standard deviation of three biological repeats. (**C**) Heterologous expression and secretion of FvRibo1 in *U. hordei* strain DS200 in planta. *Ustilago hordei* strain DS200 and DS200 expressing *FvRibo1* (encoding a secreted ribotoxin) of *Fusarium verticillioides* with *UHOR_02700* signal peptide and under the control of the *UHOR_02700* promoter (for only in planta expression) were inoculated on susceptible 12-day-old barley seedlings. Macroscopic pictures were taken at 6 dpi. Autofluorescence pictures were taken to see better cell death by using Gel-Doc (Bio-Rad). (**D**) Wheat germ agglutinin (WGA)-AF488/propidium iodide (PI) staining was performed to visualize the colonization of DS200-FvRibo1 in barley leaves compared to DS200. While green signal indicates fungal colonization, the red signal represents the plant cell walls.

To show that *U. hordei* can express and secrete functional proteins from different plant pathogenic fungi, namely *Avr4* from *Cladosporium fulvum* (a chitin-binding Avr protein that can be recognized by tomato resistance protein Cf4) and *Ribo1* (encoding a secreted ribotoxin) from *Fusarium verticillioides*, were heterologously expressed in the DS200 strain. For secretion from *U. hordei* hyphae, the open reading frames were fused with the sequence-encoding *UHOR_02700* signal peptide (SP). For constitutive expression, heterologous genes were expressed under control of the *pActin* promoter, and for specific transcriptional induction during plant colonization, the promoter *pUHOR_02700* (highly expressed in the in planta *U. hordei* effector gene) was used [20]. To confirm in vitro expression and secretion of the CfAvr4 effector protein in *U. hordei*, culture filtrates isolated from DS200-CfAvr4, DS200-FvRibo1, and DS200 strains were collected and infiltrated in *Nicotiana benthamiana* leaves expressing the *Cf4* resistance gene, a gene encoding the tomato Cf4 receptor protein, which recognizes the CfAvr4 protein and induces a hypersensitive response [44]. While neither DS200 nor DS200-FvRibo1 (expressed only in planta) culture filtrates did induce any hypersensitive-response-mediated cell death in Cf4-expressing tobacco leaves, the culture filtrate of DS200-CfAvr4 induced hypersensitive-response-mediated cell death in the presence of Cf4 (Figure 4A). Since Avr4-triggered HR cannot be observed in barley, we deployed FvRibo1 to test secretion of a functional heterologous virulence factor in planta. Heterologous expression of plant cytotoxic FvRibo1 protein in the DS200 strain was expected to negatively affect the growth of *U. hordei* on barley leaves. Macroscopic observations of infected barley leaves at 6 dpi showed that infection by the DS200 strain causes the spread of chlorosis along the leaf veins, reflecting the spread of fungal proliferation (Figure 4C). In contrast, DS200-FvRibo1-infected barley leaves displayed accumulated focal necrotic spots, reflecting restriction of fungal proliferation (Figure 4C). WGA-AF488/PI staining of infected barley leaves revealed that the DS200-FvRibo1 strain is mostly restricted to the penetration area and rarely reaches the vascular bundles, while DS200 colonized leaf veins and successfully accessed the host vascular bundles at 6 dpi (Figure 4D). In line with this, fungal biomass quantification of the DS200-FvRibo1 and DS200 strains on infected barley leaves at 6 dpi confirmed a significant virulence reduction of the DS200-FvRibo1 compared to the DS200 strain (Figure 4B). Together, these findings show that heterologous expression of FvRibo1 attenuated *U. hordei* infection (Figure 4B–D).

## 4. Discussion

Plant pathogenic smut fungi are facultative biotrophic fungal pathogens, which can infect many economically important crops, such as maize, wheat, barley, oat, and sugar cane. Recent comparative genome analysis of five plant pathogenic smut fungi, including *U. hordei*, *U. maydis*, *Sporisorium reilianum*, *Sporisorium scitamineum*, and *Melanopsichum pennsylvanicum*, showed that all of these smut fungi have relatively small genomes (about 20 Mbp) [45]. These genomic features make smut fungi excellent candidates for functional genetic and genomic approaches. The availability of complete genome assemblies, available transcriptomics data and the possibility of performing reverse genetics make the *U. hordei*–barley system a potential model for studying the molecular basis of plant–pathogen interactions. To enable and speed-up functional genetics in the *U. hordei*–barley interaction, we established several molecular tools, including a solopathogenic *U. hordei* strain, heterologous gene expression, and an efficient CRISPR/Cas9 gene editing system.

### 4.1. Establishment of a Solopathogenic Strain

The generation of haploid solopathogenic *U. hordei* strains, which express an active bE/bW heterodimer and form infectious filaments without having to fuse with a mating partner, increases the efficiency of genetic transformation by reducing the lab workload, since no duplicate mutants in opposite mating partners are needed [41]. The quantification of appressoria formation and penetration efficiency of solopathogenic strains showed that there are no significant differences compared to wild-type strains on barley leaves. This result indicates that the solopathogenic strains can be used for functional characterization of virulence factors during barley leaf colonization. Although the barley leaf infection assay did not show any difference in colonization of solopathogenic and wild-type strains, in barley seed infection assays, the solopathogenic strains rarely colonized barley inflorescence and produced teliospores compared to the wild-type strain. This observation indicates that the solopathogenic strain is only weakly pathogenic in systemic colonization of barley and may not produce all virulence factors or effectors needed at the later stages of infection. Accordingly, two generated solopathogenic *U. maydis* strains, SG200 and CL13, also showed attenuated virulence compared to wild-type strains [46,47]. This reduced virulence phenotype in solopathogenic strains could be due to having only one nucleus instead of two nuclei, which may lead to a reduced transcription level of effector genes or to a lack of the allelic variation of the two nuclei. Moreover, CL13, which is generated by replacement of only compatible *b* loci, shows a more attenuated virulence compared to the SG200 strain, which is generated by replacement of both compatible *a* and *b* loci [46,47]. In our experiments, the *U. hordei* solopathogenic strains DS199 (with only compatible *b* alleles) and DS200 (with both compatible *a* and *b* alleles) showed similar rates of virulence during barley penetration. This suggests that the presence of a compatible *a* locus is more important for the formation of infectious filaments in *U. maydis*, which has a tetrapolar mating system, than in *U. hordei*, which has a bipolar mating system [19].

Recently, Schuster et al. [48] established a CRISPR/Cas9 gene editing system for *U. maydis* with ~70% efficiency. By using a similar approach, we achieved a very efficient CRISPR/Cas9-HF based system in *U. hordei*, with ~83% gene editing efficiency in progeny. CRISPR/Cas9 gene editing of the *U. hordei Fly1* gene, a fungalysin metalloprotease involved in fungal cell separation [43], resulted in an impaired cell separation phenotype in DS200, indicating the functional conservation of this protein among smut fungi. Thus, establishment of both solopathogenic strains and a CRISPR/Cas9 gene editing system allow fast and efficient reverse genetic approaches in *U. hordei*.

### 4.2. Ultrastructural Analysis of the Ustilago hordei–Barley Biotrophic Interphase

During barley leaf colonization, *U. hordei* enters in the host cell without breaching the host plasma membrane. Thus, the *U. hordei*–barley interaction is mediated through the biotrophic interphase, which comprises the fungal cell wall (FCW), electron-dense and electron-translucent extracellular matrixes (edECM and etECM), and the plant cell wall (PCW) (in some parts of colonized tissues). The presence of vesicles in the fungal cytoplasm close to the hyphal tip and in the surrounding plant cytoplasm, as well as of plant multivesicular bodies close to fungal penetration sites, indicates that the biotrophic interphase is very active site. Some vesicles appeared to be in the process of either fusing with or pinching off the plant cell membrane. The edECM of unknown composition surrounds the FCW, and in some regions its outer surface shows irregular patterns with small protrusions. At contact sites with the PCW and at cell-to-cell penetration sites, *U. hordei* accumulates a thicker edECM, and it seems that the material causing the electron density of the ECM diffuses into the adjacent PCW. This observation indicates that the interphase between the plant cell membrane and the edECM is quite active. The plant apoplastic space contains a wide range of defense components, such as glycoside hydrolyses, proteases, peroxidases, antimicrobial proteins, and secondary metabolites, which collectively contribute to plant immunity [49]. *U. hordei* hyphae may secrete the edECM to prevent access of these plant-derived defense components to the fungal cell. Since the thickness of edECM gets higher with contact to the host PCW, one can also hypothesize that it is required for anchoring to the PCW to increase cell-to-cell penetration efficiency. Similar edECM was also observed for other smut fungi, such as the maize smut *U. maydis* and *Ustacystis waldsteiniae* (on *Waldsteinia geoides* host), rust, as well as powdery mildew (*Hyaloperonospora parasitica*) during host colonization [50,51,52,53]. Although the content of edECM remains unknown, immunogold labeling with (1-3)-β-glucan-specific antibodies revealed the presence of callose in the electron-translucent extracellular matrix (etECM). In incompatible host–pathogen interactions, callose deposition at the site of infection is a hallmark of plant immune response; however, in compatible interactions, successful pathogens (like *U. hordei*) can suppress the host defense response, including callose deposition [54]. Therefore, the presence of callose in the etECM indicates that *U. hordei* may have an ability to use callose at the biotrophic interphase as an additional carbon source. Upregulation of several 1,3 beta-glucanase encoding genes in *U. hordei* during host colonization might be involved in this process [20].

To reach the host vascular bundles, *U. hordei* grows or moves mostly intracellularly from cell to cell. Detailed observation revealed that at the site of cell-to-cell penetration, the *U. hordei* hypha developed a swollen structure, which resembled an appressorium. A similar phenotype of swollen hyphal tips was also observed in *U. maydis* during cell-to-cell penetration in maize [55]. Formation of this structure may increase the penetration efficiency of smut fungi from cell-to-cell movements. We have recently reported that *U. hordei* intracellular hyphae develop lobed haustoria-like structures during barley colonization [20]. One infected host cell could have more than one haustorium. Haustorial structures of *U. hordei* were distinguished from the normal hyphae by their bigger size and interconnected lobular shapes. While both rust and powdery mildew haustoria are formed from extracellular fungal hyphae [56,57], *U. hordei* haustoria structures originate from intracellular hyphae. In addition, a structure comparable to the neckband of rust haustoria that separates the haustorial matrix from the apoplast was not seen in our analysis [58]. Detailed transmission electron micrographs of *U. hordei* haustoria also showed that these structures contain large vacuoles with a fine granular content and intraluminal vesicles.

### 4.3. Heterologous Expression of Fungal Effectors in Ustilago hordei

Both smuts and rusts are plant pathogenic fungi belonging to the division Basidiomycotina [59]. In addition to their phylogenetic relationships, smuts (facultative biotroph) and rusts (obligate biotroph) have similar biotrophic life styles, in which they require an intimate association with their hosts to acquire nutrients and complete their pathogenic lifecycles. Apart from their phylogenetic relationships and similar lifestyles, *U. hordei*, *Bgh*, and *Pgt* also form comparable intracellular haustorial structures, secrete edECM, and infect the same host plant species. Due to host-specific adaptations during co-evolution, entirely different pathogens that share the same host can independently develop different types of effectors that interact with the same target. Accordingly, Avr2 of *C. fulvum* (fungus) [60,61,62], EPIC1 and EPIC2B of *Phytophthora infestans* (oomycete) [63], Gr-VAP1 of *Globodera rostochiensis* (nematode) [64], and Cip1 from *Pseudomonas syringae* (bacterium) [65] can interact and inhibit tomato cysteine protease, Rcr3. Therefore, the *U. hordei*–barley pathosystem allows one to perform reverse genetics for *Bgh* and *Pgt* effectors in the same host system and subsequently to identify their virulence functions.

Successful heterologous expression and secretion of the GUS-mCherry recombinant protein in *U. hordei* during barley colonization demonstrates that the designed concept is feasible. Some of the GUS-mCherry was also cleaved in the apoplastic space of barley leaf, indicating C-terminal processing of this protein. For further proof that *U. hordei* can express and secrete functional effectors from different fungi, a very robust Avr4/Cf4 pair was used to induce HR-mediated cell death. To this end, the *Avr4* avirulence gene from *C. fulvum* was expressed in DS200 in vitro. Induction of Cf4-mediated HR only in the presence of culture filtrate of the DS200-Avr4 strain indicates that DS200-Avr4 expresses and secretes functional Avr4 from a biotrophic *C. fulvum*, which can be recognized by Cf4 resistance protein. In a similar way, in planta expression and secretion of a plant cytotoxic *Ribo1* (FvRibo1) from *F. verticillioides* in DS200 was confirmed by using only in-planta-expressed *U. hordei* promoter. Heterologous expression of the FvRibo1 in DS200 negatively affects the colonization of this biotrophic smut fungus in barley. The WGA-AF488/PI staining and biomass quantification assays showed that DS200-FvRibo1 hardly moves and colonizes vascular bundles, showing significantly reduced fungal biomass compared to DS200 strain. Moreover, macroscopic and microscopic observations of focal necrotic spots on barley leaves indicated that FvRibo1 is also cytotoxic to barley cells. Heterologous expression of exogenous effector genes from different plant pathogenic fungi showed that the solopathogenic *U. hordei* DS200 strain can be used for functional characterization of effectors from biotrophic phytopathogens as well. Moreover, by comparing native expression patterns of effectors of interest with recently published transcriptome data for *U. hordei* [20], one can determine the most suitable *U. hordei* promoters for heterologous gene expression resembling native gene expression levels.

The biotrophic infection of barley leaves, formation of intracellular hyphae and haustorial structures, as well as the molecular tools presented in this study make the *U. hordei* pathosystem a useful platform for the functional analysis of effector proteins from biotrophic fungi.

## Data Availability

Data is available in this article and as Supplementary Material.

## Supplementary Materials

The following are available online at https://www.mdpi.com/2309-608X/7/2/86/s1, Figure S1: Ustilago hordei-barley infection assay, Figure S2: Cell-to-cell penetration of Ustilago hordei, Figure S3: Ultrastructural features of the Ustilago hordei during barley colonization, Table S1: Plasmids and primers used in this study.
